# Taxanes and platinum derivatives impair Schwann cells via distinct mechanisms

**DOI:** 10.1038/s41598-017-05784-1

**Published:** 2017-07-20

**Authors:** Satoshi Imai, Madoka Koyanagi, Ziauddin Azimi, Yui Nakazato, Mayuna Matsumoto, Takashi Ogihara, Atsushi Yonezawa, Tomohiro Omura, Shunsaku Nakagawa, Shuji Wakatsuki, Toshiyuki Araki, Shuji Kaneko, Takayuki Nakagawa, Kazuo Matsubara

**Affiliations:** 1 Department of Clinical Pharmacology and Therapeutics, Kyoto University Hospital, 54 Shogoin-Kawahara-cho, Sakyo-ku, Kyoto 606-8507 Japan; 2 Department of Molecular Pharmacology, Graduate School of Pharmaceutical Sciences, Kyoto University, 46-29 Yoshida-Shimoadachi-cho, Sakyo-ku, Kyoto 606-8501 Japan; 3 Department of Biochemistry, Faculty of Pharmacy, Kabul University, Jamal Mina, Aliabad, Kabul, Afghanistan; 4 Department of Peripheral Nervous System Research, National Institute of Neuroscience, National Center of Neurology and Psyhicatry, 4-1-1 Ogawa-Higashi, Kodaira, Tokyo 187-8502 Japan

## Abstract

Impairment of peripheral neurons by anti-cancer agents, including taxanes and platinum derivatives, has been considered to be a major cause of chemotherapy-induced peripheral neuropathy (CIPN), however, the precise underlying mechanisms are not fully understood. Here, we examined the direct effects of anti-cancer agents on Schwann cells. Exposure of primary cultured rat Schwann cells to paclitaxel (0.01 μM), cisplatin (1 μM), or oxaliplatin (3 μM) for 48 h induced cytotoxicity and reduced myelin basic protein expression at concentrations lower than those required to induce neurotoxicity in cultured rat dorsal root ganglion (DRG) neurons. Similarly, these anti-cancer drugs disrupted myelin formation in Schwann cell/DRG neuron co-cultures without affecting nerve axons. Cisplatin and oxaliplatin, but not paclitaxel, caused mitochondrial dysfunction in cultured Schwann cells. By contrast, paclitaxel led to dedifferentiation of Schwann cells into an immature state, characterized by increased expression of p75 and galectin-3. Consistent with *in vitro* findings, repeated injection of paclitaxel increased expression of p75 and galectin-3 in Schwann cells within the mouse sciatic nerve. These results suggest that taxanes and platinum derivatives impair Schwan cells by inducing dedifferentiation and mitochondrial dysfunction, respectively, which may be important in the development of CIPN in conjunction with their direct impairment in peripheral neurons.

## Introduction

Taxanes and platinum derivatives are effective first-line chemotherapy agents. However, up to 50% of patients receiving these anti-cancer agents develop a dose-limiting side effect: chemotherapy-induced peripheral neuropathy (CIPN). Symptoms include paresthesia, dysesthesia, numbness, loss of balance, and muscle weakness^1–3^. To date, there is no effective way of preventing and/or treating CIPN, which can become chronic and persist for months or years after termination of chemotherapy^4, 5^.

Several animal models of CIPN have been developed to examine the causal mechanisms. Early morphological studies have provided evidence that paclitaxel induces distal axonopathy after systemic administration at relatively high doses or after local injection directly into a peripheral nerve^6, 7^. Based on these results, taxane-induced peripheral neuropathy has been believed to be secondary to taxane-induced inhibition of the dynamic assembly and disassembly of β-tubulin, resulting in a progressive distal axonopathy^8–11^. However, growing evidence suggests an alternative hypothesis^7, 12–14^. For example, electron microscopic studies of rat peripheral nerves show that treatment with low dose paclitaxel causes a painful peripheral neuropathy, but fails to induce axonal degeneration in peripheral nerves. On the other hand, platinum derivatives such as cisplatin and oxaliplatin exert cytotoxic effects in the dorsal root ganglia (DRG) neurons, which are mediated via formation of inter- and intra-strand crosslinks in DNA, and accumulation of platinum-mitochondrial DNA adducts^15, 16^. However, it is suggested that the impairment of satellite cells and Schwann cells or glial activation in the spinal cord, as well as DRG sensory neurons, are also involved in the pathogenesis of platinum derivative-induced neuropathy^17^. Thus, the complex machinery underlying CIPN pathogenesis remains unclear and is the subject of much debate.

Schwann cells are peripheral nervous system glial cells that form a thin myelin sheet by tightly wrapping around axons to enable rapid saltatory conduction of action potentials^18, 19^. A growing body of evidence suggests that Schwann cells play a crucial role in the outgrowth and guidance of regrowing peripheral axons after injury. Immediately after peripheral nerve injury, Schwann cells in the injured area transdifferentiate and migrate to the distal end to form a denervated Schwann cell band^20, 21^. The growth cone of a regrowing peripheral nerve fiber advances toward its original target using the Schwann cells as a guide. Thus, Schwann cells play a major supportive role in the maintenance of the peripheral nervous system, raising the intriguing possibility that impairment of Schwann cells and consequent disruption of intercellular interactions between myelin-forming mature Schwann cells and axons by anti-cancer agents may be important for the pathogenesis of CIPN. Based on this hypothesis, the present study was designed to ascertain the direct effect of anti-cancer agents (paclitaxel, cisplatin and oxaliplatin) on primary Schwann cell cultures and on myelin-forming Schwann cells in the mouse sciatic nerve.

We show for the first time that treatment with paclitaxel induces the dedifferentiation of myelin-forming Schwann cells, whereas cisplatin and oxaliplatin induced cytotoxicity accompanied by mitochondrial dysfunction at concentrations lower than those required to impairment of DRG neurons. The present data suggest that these direct effects of paclitaxel, cisplatin and oxaliplatin on Schwann cells (as well as a their direct toxicity in peripheral neurons) might be the underlying cause of CIPN.

## Results

### Differentiation of cultured rat immature Schwann cells

**Figure 1:**
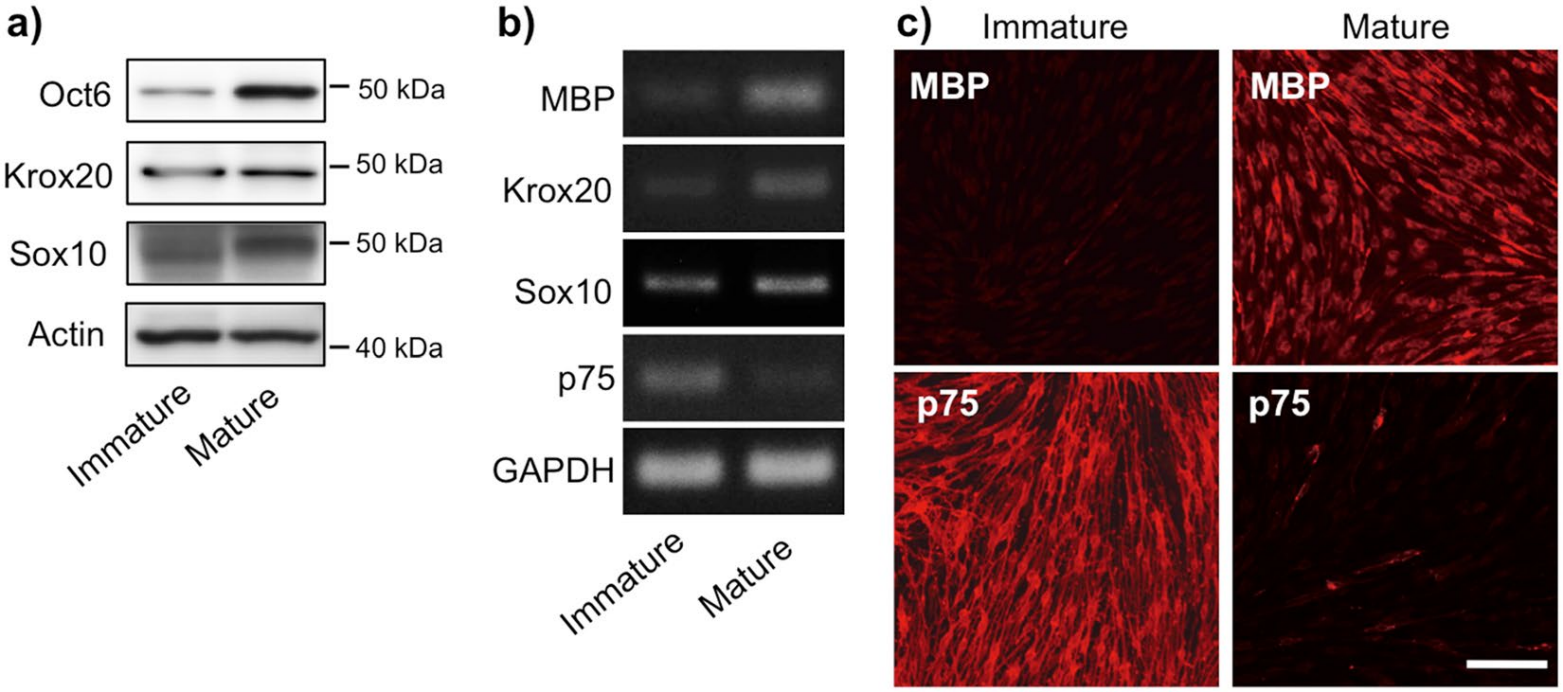
Differentiation of primary cultured Schwann cells obtained from the sciatic nerves of neonatal rat pups. (**a**) A representative Western blot showing specific lineage markers (Oct6, Krox20, and Sox10) in Schwann cells just before (*left lane*) and 2 days after (*right lane*) differentiation. Actin is shown as an internal control. (**b**) Representative RT-PCR showing expression of MBP, Krox20, SOX10 and p75 mRNA in Schwann cells just before (*left lane*) and 2 days after (*right lane*) differentiation. GAPDH is shown as an internal control. Full images of Western blot (**a**) and RT-PCR (**b**) are shown in Supplementary Figs S1 and S2, respectively. (**c**) Immunofluorescent staining for MBP (*upper*) and p75 (*lower*) in Schwann cells just before (*left*) and 2 days after (*right*) differentiation. Scale bar: 100 μm. A mature cell phenotype is characterized by induced expression of MBP, Oct6 and Krox20, and loss of p75 expression. All experiments were conducted using at least three independent samples.

We utilized cultured primary Schwann cells from the sciatic nerves of neonatal rats to evaluate the direct effect of anti-cancer agents. After 2 days of culture in differentiation medium, Schwann cells showed a differentiated cell phenotype, characterized by increased expression of pro-myelinating transcription factor Oct6 protein, myelinating regulator transcription factor Krox20 protein and mRNA (Figs 1a and b, Supplementary Figs S1 and S2), and the major myelin proteins MBP protein and mRNA (Fig. 1b,c and Supplementary Fig. S2). Before cell differentiation, there was little or no expression of these Schwann cell markers. Consistent with previous reports showing that Sox10 is a marker constitutively expressed throughout Schwann cell differentiation^22, 23^, we observed expression of Sox10 protein and mRNA on both immature and differentiated Schwann cells (Fig. 1a,b, Supplementary Figs S1 and S2). We further confirmed sufficient induction of Schwann cell differentiation by noting almost complete disappearance of both immunoreactivity (IR) and mRNA expression of an immature Schwann cell marker, p75 neurotrophin receptor (Fig. 1b,c and Supplementary Fig. S2).

### Anti-cancer agents reduce the viability of cultured Schwann cells

**Figure 2:**
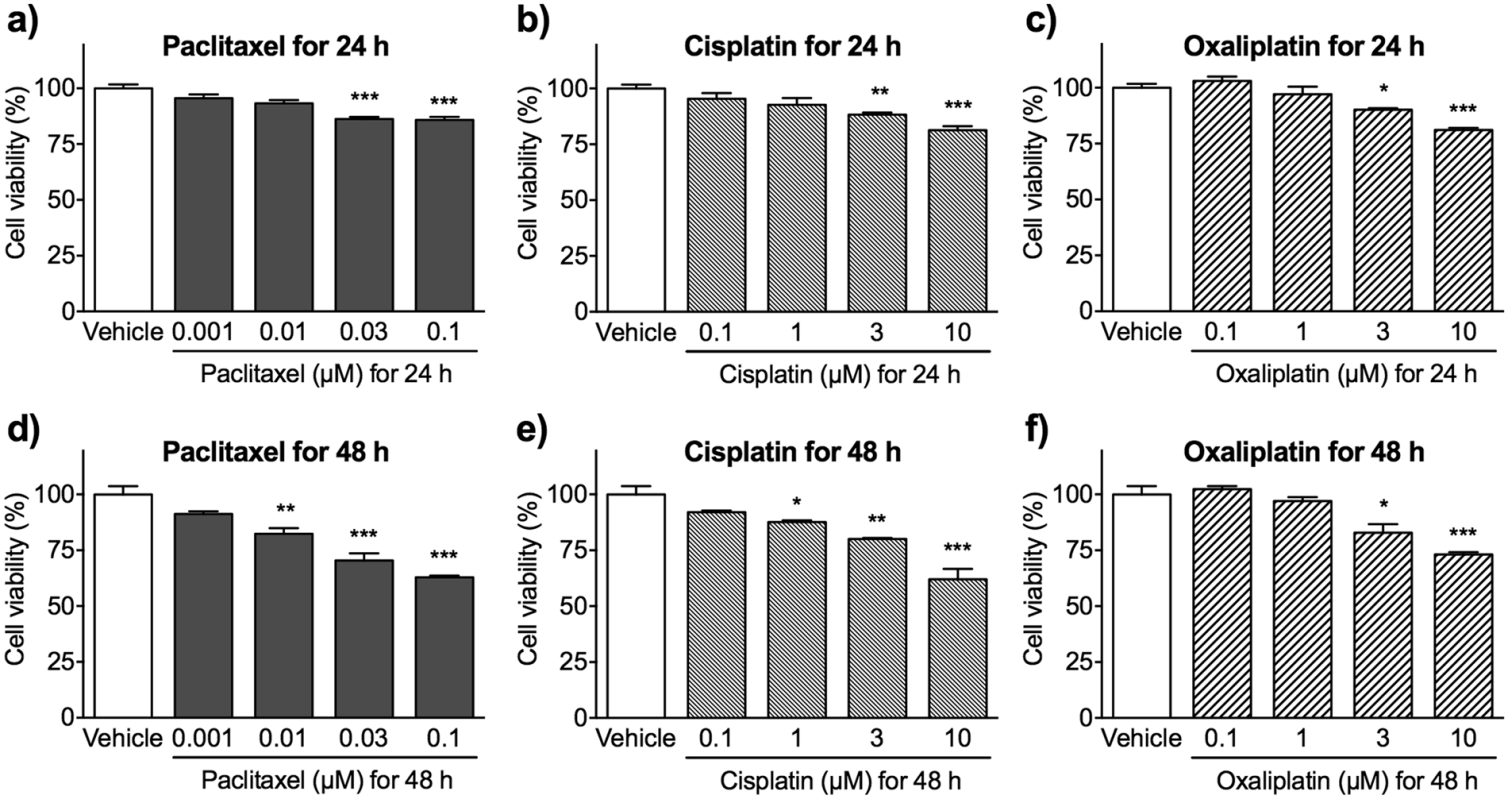
Viability of Schwann cells after exposure to paclitaxel, cisplatin or oxaliplatin. At 2 days after culture in differentiation medium, Schwann cells were treated with vehicle (0.1% DMSO), paclitaxel (**a**,**d**; 0.001–0.1 μM), cisplatin (**b**,**e**; 0.1–10 μM) or oxaliplatin (**c**,**f**; 0.1–10 μM) for 24 h (**a**–**c**) or 48 h (**d**–**f**). Cell viability was measured in an MTT assay, and the results were expressed as a percentage relative to vehicle-treated cells. Each column represents the mean ± S.E.M. *n* = 3–6. *p < 0.05, **p < 0.01, and ***p < 0.001 *vs*. the vehicle-treated group.

We next evaluated the effects of anti-cancer agents on the viability of differentiated Schwann cells using a 3-(4,5-dimethyldiazol-2-yl)-2,5-diphenyltetrazolium bromide (MTT) assay. Each of the anti-cancer agents reduced the viability of Schwann cells in a concentration-dependent manner after either 24 or 48 h of treatment (Fig. 2). The number of viable cells detected following 24 h exposure to paclitaxel, cisplatin or oxaliplatin (0.03, 3 or 3 μM, respectively) was significantly lower than after exposure to vehicle (Fig. 2a–c). Paclitaxel, cisplatin and oxaliplatin (0.01, 1 and 3 μM, respectively) showed significant cytotoxic effects against Schwann cells after 48 h (Fig. 2d–f). Treatment for 48 h tended to be more toxic than treatment for 24 h. Thus, 0.01 μM paclitaxel, 1 μM cisplatin and 3 μM oxaliplatin were taken to be the minimum concentrations showing a significant effect against Schwann cells.

### Paclitaxel, but not cisplatin and oxaliplatin, induces morphological changes in cultured Schwann cells

**Figure 3:**
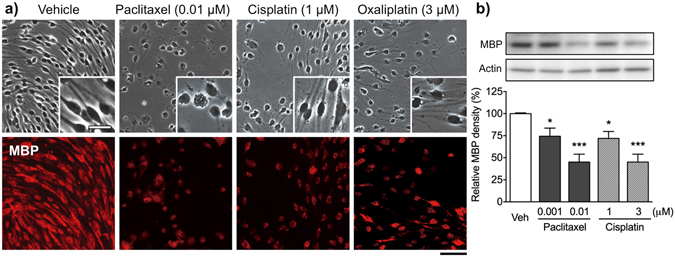
Effects of paclitaxel, cisplatin and oxaliplatin on Schwann cell morphology and MBP expression. (**a**) Phase (*upper*) and fluorescence (*lower*) micrographs of differentiated Schwann cells labeled with an anti-MBP antibody. Differentiated Schwann cells were treated with vehicle (0.1% DMSO), paclitaxel (0.01 μM), cisplatin (1 μM) or oxaliplatin (3 μM) for 48 h. Cisplatin and oxaliplatin reduced the number of MBP-positive Schwann cells, but did not affect cell morphology. Unlike cisplatin and oxaliplatin, paclitaxel induced morphological changes, characterized by retraction of bipolar processes and a rounded shape. Scale bar: 100 μm (25 μm in enlarged images). (**b**) Expression of MBP in Schwann cells 48 h after treatment with vehicle (0.1% DMSO), paclitaxel (0.001 and 0.01 μM) or cisplatin (1 and 3 μM) was analyzed by Western blotting. (*Upper panels*) Representative Western blot showing expression of MBP and actin. (*Lower panels*) Quantification of band intensity. The intensity of each band was normalized to that of actin (loading control). Each column represents the mean ± S.E.M. *n* = 5–8. *p < 0.05 and ***p < 0.001 *vs*. the vehicle-treated group. Images of the full blots are shown in Supplementary Fig. S3.

To gain further insight into the direct effects of anti-cancer agents on Schwann cells, we examined the effects of paclitaxel, cisplatin and oxaliplatin on the morphology of differentiated Schwann cells (Fig. 3a). Differentiated cells had a typical Schwann cell phenotype, being bipolar and spindle shaped, with a small cytoplasm. Cells also stained positive for MBP. Treatment with paclitaxel (0.01 μM), cisplatin (1 μM) or oxaliplatin (3 μM) for 48 h reduced the number of MBP-positive Schwann cells. Expression of MBP was significantly lower after treatment with paclitaxel (0.001 and 0.01 μM) or cisplatin (1 and 3 μM) in a concentration-dependent manner (Fig. 3b and Supplementary Fig. S3). Unlike cisplatin and oxaliplatin, paclitaxel caused marked morphological changes, characterized by retraction of bipolar processes and a rounded shape (Fig. 3a).

### Anti-cancer agents induce impairment of myelin formation in a mixed culture of Schwann cells and DRG neurons

**Figure 4:**
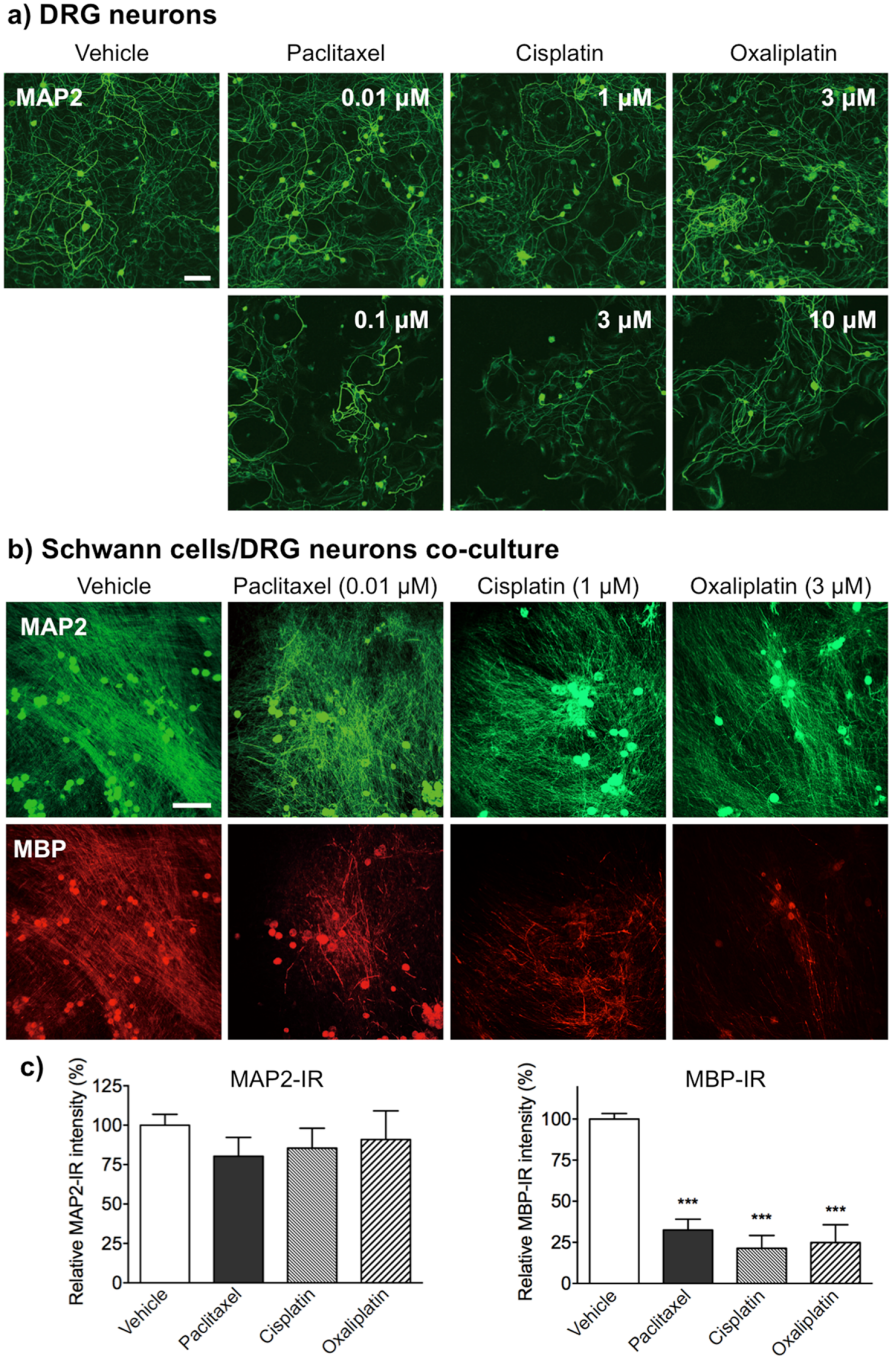
Effects of paclitaxel, cisplatin and oxaliplatin on primary cultured DRG neurons or on co-cultures of Schwann cells and DRG neurons. (**a**) Immunofluorescence staining of primary cultured DRG neurons for MAP2. Primary cultured DRG neurons were treated with vehicle (0.1% DMSO), paclitaxel (0.01 and 0.1 μM), cisplatin (1 and 3 μM) or oxaliplatin (3 and 10 μM) for 48 h. Only higher doses of paclitaxel (0.1 μM), cisplatin (3 μM) and oxaliplatin (10 μM) reduced the number of DRG neurons and associated dendritic trees, and shortened the neuritic processes. Scale bar: 100 μm. (**b**) Co-cultures were treated with vehicle (0.1% DMSO), paclitaxel (0.01 μM), cisplatin (1 μM), or oxaliplatin (3 μM) for 48 h. Scale bar: 200 μm. Each treatment reduced formation of MBP-positive myelin; however, no morphological changes were observed in MAP2-positive DRG neurons. (**c**) MBP- or MAP2-IR in co-cultures. Each column represents the mean ± S.E.M. *n* = 3. ***p < 0.001 *vs*. the vehicle-treated group.

The direct effects of anti-cancer agents on the viability and morphology of rat primary DRG neurons were also investigated (Fig. 4a). Primary DRG neurons stained with an antibody specific for the neuronal marker microtubule-associated protein-2 (MAP2) were spherical, with multi-neuritic processes. Treatment with paclitaxel (0.01 μM), cisplatin (1 μM) or oxaliplatin (3 μM) for 48 h did not affect the viability or morphology of primary DRG neurons. By contrast, exposure to the three drugs at higher concentrations (paclitaxel, 0.1 μM; cisplatin, 3 μM; and oxaliplatin, 10 μM) reduced the number of neurons and their dendritic trees, and shortened the length of the neuritic processes, when compared with vehicle treatment.

The next study was undertaken to determine whether treating Schwann cell/DRG neuron co-cultures with anti-cancer agents leads to demyelination. In a mixed culture of Schwann cells and DRG neurons, Schwann cells differentiated along the DRG axons and eventually formed MBP-positive myelin segments, as indicated by the apparent overlap of MAP2- and MBP-IR (Supplementary Fig. S4). Treatment with paclitaxel (0.01 μM), cisplatin (1 μM) or oxaliplatin (3 μM) for 48 h led to a significant reduction in MBP-IR, suggesting loss of myelinating Schwann cells in the co-cultures. However, treatment did not affect MAP2-positive neural axons and MAP2-IR (Fig. 4b and c).

### Cisplatin and oxaliplatin, but not paclitaxel, cause mitochondrial dysfunction in cultured Schwann cells

**Figure 5:**
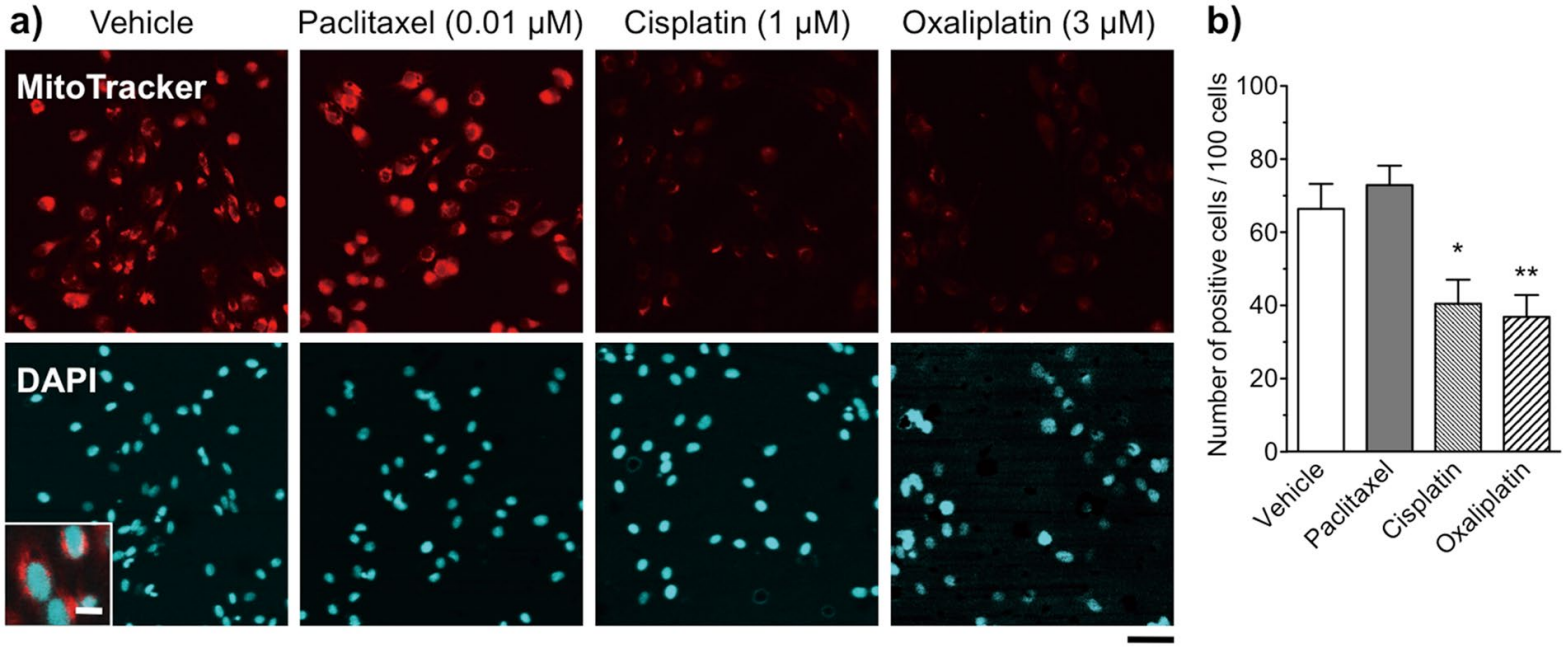
Changes in mitochondrial activity in Schwann cells after treatment with paclitaxel, cisplatin or oxaliplatin. (**a**) Fluorescence micrographs of Schwann cells labeled with the MitoTracker probe (red) and DAPI (blue). Schwann cells were treated with vehicle (0.1% DMSO), paclitaxel (0.01 μM), cisplatin (1 μM) or oxaliplatin (3 μM) for 48 h. Scale bar: 50 μm (10 μm in enlarged images) in the vehicle-treated group. (**b**) Number of mitochondria-labeled cells per 100 DAPI-positive cells. The intensity and number of fluorescently labeled mitochondria were reduced after treatment with either cisplatin or oxaliplatin, but not after treatment with paclitaxel. Each column represents the mean ± S.E.M. *n* = 12. *p < 0.05 and **p < 0.01 *vs*. the vehicle-treated group.

Cisplatin directly binds to both mitochondrial DNA and nuclear DNA, thereby inducing dysfunction, vacuolization, and degradation of mitochondria in DRG neurons^24^. To determine whether anti-cancer agents induce mitochondrial dysfunction in cultured Schwann cells, we performed fluorescence microscopic analyses using the Δψ_m_-sensitive probe MitoTracker Red (Fig. 5). This probe used for mitochondria labeling is a derivative of X-Rosamine, which fluoresces upon oxidation, passively diffuses across the plasma membrane due its lipophilic nature and accumulates in functional mitochondria driven by Δψ_m_^25^. Images shown in Fig. 5a (merged image shown for the vehicle-treated group) indicate that mitochondria are preferentially confined at the perinuclear region of Schwann cells. Treatment with paclitaxel (0.01 μM) for 48 h (the same concentration that caused marked morphological changes in Schwann cells) had no effect on the intensity of immunofluorescent labeling of mitochondria. By contrast, the number of stained mitochondria was markedly reduced after exposure to cisplatin (1 μM) or oxaliplatin (3 μM) for 48 h. The total number of mitochondria-labeled cells per 100 4,6-diamidino-2-phenylindole (DAPI)-positive cells observed after treatment with either cisplatin or oxaliplatin for 48 h was significantly lower than that observed in the vehicle control group, whereas there was no difference between the vehicle- and paclitaxel-treated groups (Fig. 5b). We could not detect any changes in the morphology and the number of DAPI-stained mitochondria in cisplatin- or oxaliplatin-treated Schwann cells. The decreased fluorescence of Mitotracker in Schwann cells after the treatment with platinum derivatives may be due to the loss of membrane potential, rather than mitochondrial degradation.

### Taxanes induces dedifferentiation of cultured Schwann cells

**Figure 6:**
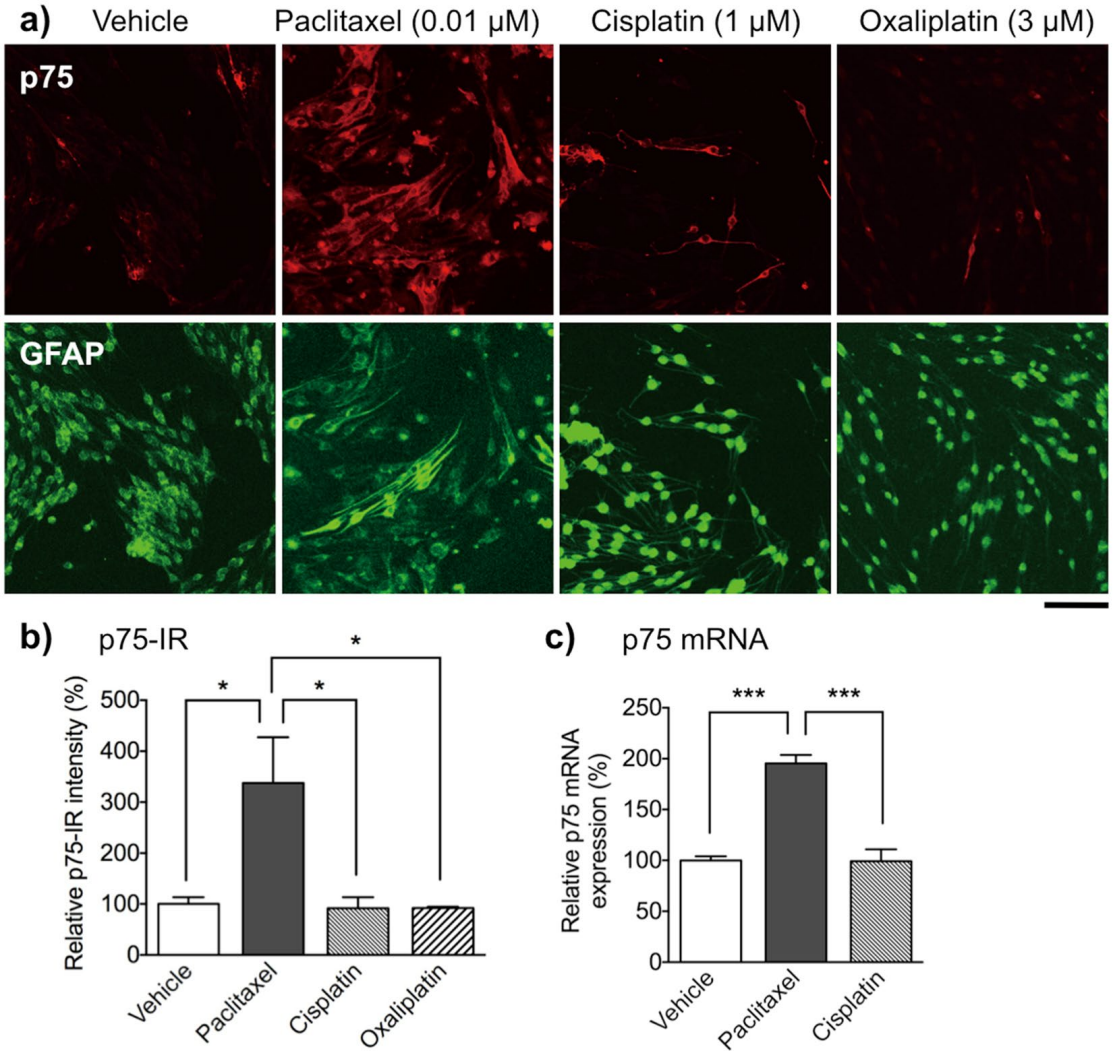
Effect of paclitaxel, cisplatin and oxaliplatin on expression of p75 in primary cultured Schwann cells. Schwann cells were treated with vehicle (0.1% DMSO), paclitaxel (0.01 μM), cisplatin (1 μM) or oxaliplatin (3 μM) for 48 h. (**a**) Immunofluorescent staining of primary cultured Schwann cells for p75 (red) and GFAP (green). Scale bar: 100 μm. (**b**) p75-IR in Schwann cells. Each column represents the mean ± S.E.M. *n* = 3. ***p < 0.001 *vs*. the vehicle-treated group. Only weak p75-IR was observed in GFAP-positive Schwann cells after treatment with vehicle, cisplatin, or oxaliplatin, whereas paclitaxel increased p75-IR in GFAP-positive Schwann cells. (**c**) Quantitative real-time PCR analysis of p75 mRNA expression in Schwann cells. Each column represents the mean ± S.E.M. *n* = 3. ***p < 0.001. Treatment with paclitaxel, but not cisplatin, led to a significantly increased in expression of p75 mRNA.

**Figure 7:**
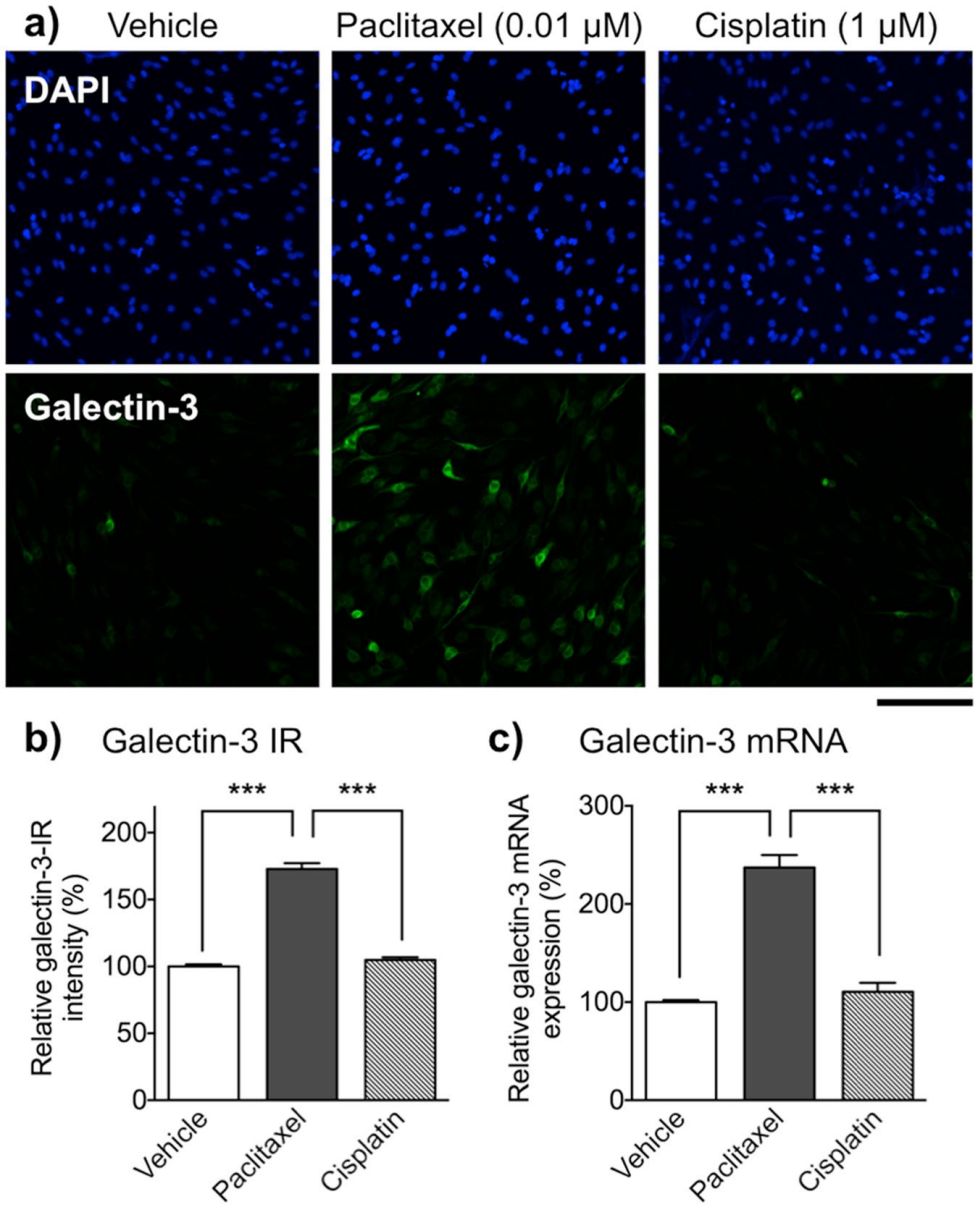
Effect of paclitaxel, cisplatin and oxaliplatin on expression of galectin-3 in primary cultured Schwann cells. Schwann cells were treated with vehicle (0.1% DMSO), paclitaxel (0.01 μM) or cisplatin (1 μM) for 48 h. (**a**) Immunofluorescence staining of primary cultured Schwann cells with DAPI (blue) and an anti-galectin-3 antibody (green). Scale bar: 100 μm. (**b**) Galectin-3-IR in Schwann cells. Each column represents the mean ± S.E.M. *n* = 3. ***p < 0.001 *vs*. the vehicle-treated group. Only weak galectin-3-IR was observed in Schwann cells after treatment with vehicle or cisplatin, whereas paclitaxel increased galectin-3-IR in Schwann cells. (**c**) Quantitative real-time PCR analysis of galectin-3 mRNA expression in Schwann cells. Each column represents the mean ± S.E.M. *n* = 3. ***p < 0.001. Treatment with paclitaxel, but not cisplatin, led to a significant increase in expression of galectin-3 mRNA.

To better understand the direct effect of paclitaxel on Schwann cells, we focused on the expression pattern of p75 and galectin-3, markers of immature and dedifferentiated Schwann cell, respectively. Fluorescence microscopic analyses revealed only weak p75-IR but robust glial fibrillary acidic protein (GFAP)-IR (a typical Schwann cell marker) in Schwann cells following a 48 h exposure to cisplatin (1 μM) or oxaliplatin (3 μM), a pattern similar to that observed in the vehicle-treated group. By contrast, treatment with paclitaxel (0.01 μM) for 48 h led to a significant increase in p75-IR in GFAP-immunopositive Schwann cells when compared with that in the vehicle-, cisplatin- or oxaliplatin-treated group (Fig. 6a and b). Expression of p75 mRNA was also significantly higher in the paclitaxel-treated group than in the vehicle-treated group, whereas cisplatin (1 µM) had no effect (Fig. 6c). Similarly, galectin-3-IR and mRNA expression was significantly increased in Schwann cells treated with paclitaxel (0.01 μM) for 48 h, whereas cisplatin (1 μM) had no effect (Fig. 7). To evaluate the relationship between the inhibition of the dynamic assembly/disassembly of β-tubulin by paclitaxel and Schwann cell dedifferentiation, we examined the effects of docetaxel (another taxane derivatives) or vincristine (an inhibitor of tubulin assembly) on Schwann cells. Like paclitaxel, treatment with docetaxel (0.001–0.1 μM) or vincristine (0.001–0.1 μM) for 48 h reduced Schwann cell viability in a concentration-dependent manner. Under these conditions, both docetaxel (0.01 μM) and vincristine (0.01 μM) induced an increase in p75- and galectine-3-IR in Schwann cells, a pattern similar to that observed in the paclitaxel-treated group (Supplementary Fig. S5).

### Paclitaxel causes Schwann cell dedifferentiation in the mouse sciatic nerves

**Figure 8:**
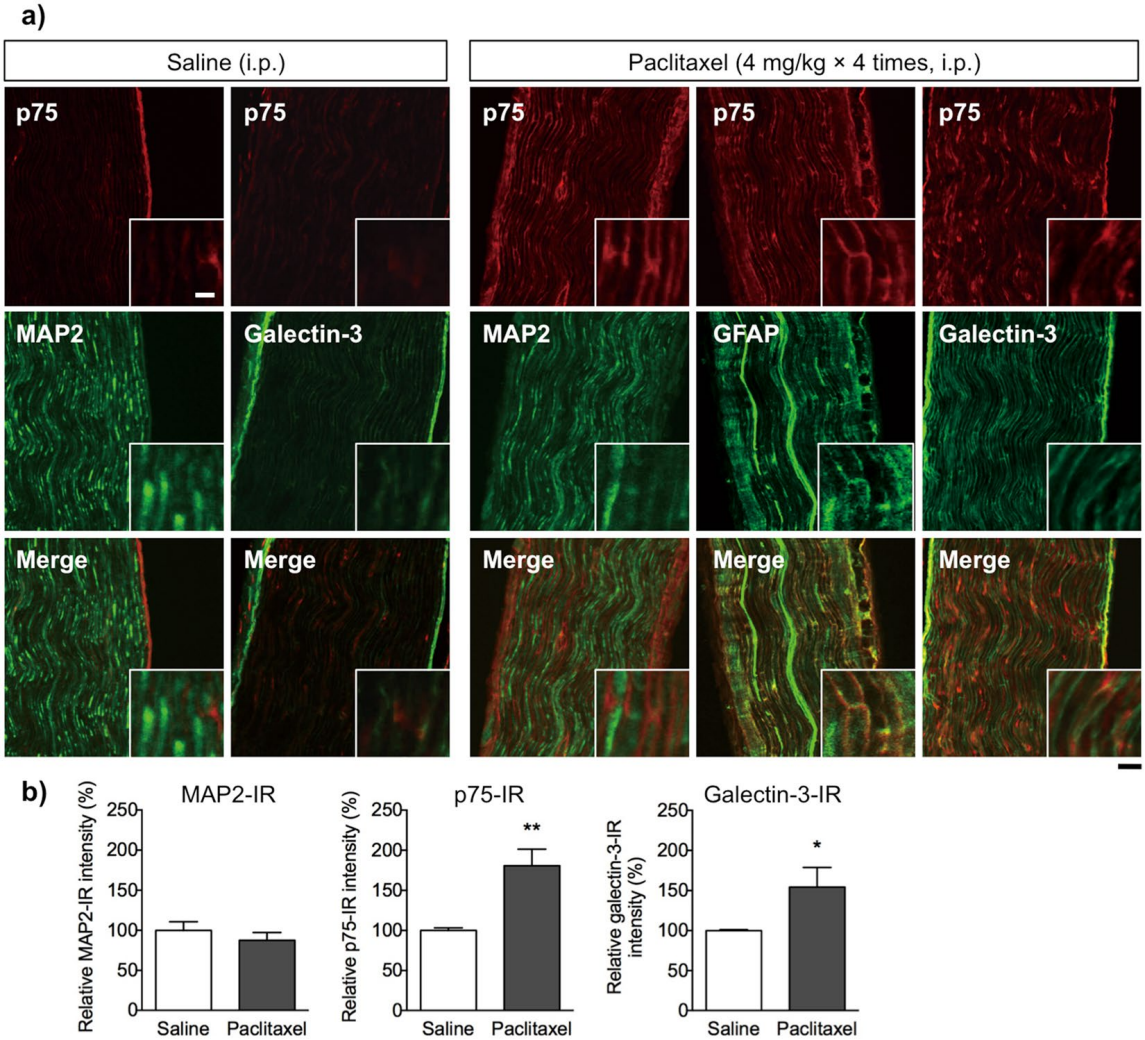
Changes in the expression of p75 and galectin-3 in sciatic nerves isolated from mice treated with paclitaxel. Paclitaxel (4 mg/kg) was injected intraperitoneally on Days 0, 3, 5, and 7. At 1 day after the last injection, mice were fixed and subjected to immunohistochemical assay. (**a**) Longitudinal sections of sciatic nerve isolated from saline- or paclitaxel-treated mice were co-immunolabeled for p75 (red), or MAP2, GFAP or galectin-3 (green). Scale bar: 50 μm (10 μm in enlarged images). Repeated injection of paclitaxel increased p75-IR in GFAP- or galectin-3-positive Schwann cells surrounding MAP2-positive neural axons. (**b**) Quantification of the intensity of fluorescence associated with MAP2, p75 or galectin-3. Each column represents the mean ± S.E.M. *n* = 3–5. *p < 0.05 and **p < 0.01 *vs*. the vehicle-treated group.

When mice were repeatedly treated intraperitoneal (i.p.) with paclitaxel (4 mg/kg × 4), the 50% paw withdrawal threshold in response to tactile stimuli decreased gradually at 4 and 7 days after the first injection (Supplementary Fig. S6). Under these conditions, p75-IR in sciatic nerves of mice treated with paclitaxel was also examined (Fig. 8a). Repeated injection of paclitaxel increased p75-IR in the longitudinal section of the sciatic nerve, although only weak p75-IR was detected in the same section from saline-treated mice. The p75-immunolabeling pattern appeared to surround MAP2-positive neural axons, and was localized in GFAP-positive Schwann cells. Consistent with this, galectin-3-IR in longitudinal sections of sciatic nerve from paclitaxel-treated mice also increased. Galectin-3-IR in the sciatic nerve of paclitaxel-treated mice was highly co-localized in GFAP-positive Schwann cells, whereas only weak galectin-3-IR was detected in the sciatic nerve of saline-treated mice. Quantitative analysis revealed that p75- and galectin-3-IR in the sciatic nerve of paclitaxel-treated mice was significantly higher than that in saline-treated mice, however, there was no difference in MAP2-IR between paclitaxel- and saline-treated mice (Fig. 8b).

## Discussion

Here, we showed for the first time that paclitaxel induced dedifferentiation of myelin-forming Schwann cells with their morphological changes, whereas both cisplatin and oxaliplatin induced cell toxicities in Schwann cells accompanied with mitochondrial dysfunction at concentrations lower than those required to induce neurotoxicity.

Schwann cell precursors, which represent the first transitional stage of the Schwann cell lineage, differentiate into immature Schwann cells, which then give rise to pro-myelinating Schwann cells. These cells eventually develop into mature myelin-forming Schwann cells^26^. Schwann cells express specific lineage markers at each stage of differentiation^27^. Consistent with previous studies, we found that Schwann cells dissociated from the sciatic nerve showed an immature phenotype, with low expression of a myelin-associated molecule (MBP) and myelinating transcription factors (Oct6 and Krox20), and high expression of p75^18, 28^. We also observed accelerated transformation of Schwann cells into differentiated Schwann cells when cultured in differentiation medium, characterized by almost complete disappearance of p75 expression and increased expression of MBP, Oct6 and Krox20^29, 30^.

We showed that *in vitro* treatment with paclitaxel, cisplatin or oxaliplatin at clinically relevant plasma concentrations^31–33^ had clear and direct effects on primary cultured Schwann cells. Previous reports suggest that mitochondrial dysfunction and subsequent oxidative stress in peripheral neurons are essential mechanisms underlying platinum derivative-induced CIPN^34^. However, the present results indicate that cisplatin and oxaliplatin have direct cytotoxic effects on Schwann cells without inducing morphological changes, and that these effects are accompanied by mitochondrial dysfunction at drug concentrations lower than those required to induce neurotoxicity. Like platinum derivatives, *in vitro* treatment with paclitaxel induced direct cytotoxic effects against Schwann cells, but caused dynamic morphological changes without any mitochondrial dysfunction in Schwann cells at relatively lower concentration (0.01 μM). By contrast, a previous report shows that *in vitro* treatment with paclitaxel causes mitochondrial dysfunction in both cultured neurons and Schwann cells^35^. In that report, a higher concentration of paclitaxel (approximately 0.29 μM) was applied to neural and Schwann cell cultures, whereas we used paclitaxel at 0.01 μM (the minimum concentrations showing a significant effect against Schwann cells) to minimize its cytotoxic effects. Thus, it seems likely that lower concentrations of paclitaxel can induce morphological changes in Schwann cells prior to mitochondrial dysfunction.

It should be noted that the treatment with relatively low concentrations of paclitaxel, cisplatin and oxaliplatin reduced myelin-forming Schwann cells in the Schwann cell/DRG neuron co-cultures without causing damages to the neurons. Furthermore, repeated treatment with paclitaxel had no meaningful effect on the MAP2-positive axons of sciatic nerves from mice with mechanical allodynia. Thus, the present findings provide evidence that Schwann cells might be a candidate target of these anti-cancer agents that induce CIPN. However, previous light and electron microscopy studies demonstrate that no severe demyelination of peripheral nerve axons occurs in rats after the weekly i.p. injection of cisplatin or paclitaxel for 4–5 weeks^36, 37^. It is widely accepted that myelin-forming Schwann cells participate in the maintenance and repair of peripheral nerves via direct cell-cell interaction with axons or via release of neurotrophins^18, 19, 21^. A reduction of myelin-forming Schwann cells causes neurological dysfunction or abnormal neural conduction, both of which may underlie the pathogenesis of diabetic neuropathy^38^ and Charcot-Marie-Tooth disease^39^. Taken together, the present findings suggest that platinum derivatives- or paclitaxel-induced impairment of myelin-forming Schwann cells with mitochondrial dysfunction or dynamic morphological changes, respectively, seem likely to result in the myelin instability and reduction in myelin sheath integrity. Analysis of nerve action potential recordings in rodents suggested that multiple injection of paclitaxel reduces the peak amplitude and conductance velocity^40, 41^. In addition, the reduction of the peak amplitude is observed in humans treated with paclitaxel^42^. Reduced nerve conductance velocity is also documented in rats treated with oxaliplatin^43^. It is possible that functional impairment of peripheral neurons may be due to myelin instability and a reduction of myelin sheath integrity induced by the anti-cancer agents.

On the other hand, Peters *et al*. report that expression of a stress-inducible gene, ATF3, in rat DRG neurons increases at 4 to 10 days after intravenous administration of paclitaxel, whereas ATF3 expression in Schwann cells is not observed until Day 6 post-infusion, suggesting that paclitaxel preferentially affects DRG neurons^44^. The results presented herein show that lower concentrations of anti-cancer agents directly affect Schwann cells, although we never clarified its temporal and functional effects on DRG neurons. Thus, our present data do not exclude the possibility that preferential impairment of peripheral neurons by the anti-cancer agents is responsible for CIPN. Nevertheless, the present data suggest that the direct effects of anti-cancer agents on Schwann cells might contribute to the pathogenesis of CIPN in conjunction with their direct impairment of peripheral neurons. Further experiments will be needed to validate the current *in vitro* findings by using animal models of CIPN following repeated administration of anti-cancer agents, just like clinical treatment protocols.

The key finding of this study is that treatment with paclitaxel, but not platinum derivatives, causes dedifferentiation of Schwann cells, as indicated by increased expression of an immature Schwann cell marker, p75, increased expression of a dedifferentiated Schwann cell marker, galectin-3 (also known as macrophage-2 antigen), and reduced expression of a differentiated Schwann cell marker, MBP. We also showed increased expression of p75 and galectin-3 in Schwann cells surrounding the neural axon in sciatic nerves isolated from mice with paclitaxel-induced peripheral neuropathy. Myelinating Schwann cells can transform into an immature phenotype and re-enter the cell cycle to support nerve regeneration in response to peripheral nerve injury^45^. p75 is induced in demyelinating Schwann cells during Wallerian degeneration and regrowth of peripheral axons, and is involved in Schwann cell dedifferentiation^46–48^. Dedifferentiated Schwann cells also show high expression of galectin-3, which is not detectable in immature Schwann cells and plays an important role in lectin-mediated phagocytosis of degraded material at the site of injury^49, 50^. Thus, to the best of our knowledge, the present data are the first to suggest that paclitaxel induces Schwann cells to dedifferentiate into an immature state, which is akin to Schwann cell transformation after the nerve injury. Recently, Nobusue *et al*. proposed a new mechanism to explain the relationship between cytoskeletal dynamics and cellular differentiation of adipocytes^51^. They suggest that depolymerization of actin fibers in adipocytes triggers the differentiation program, which is mediated by increased expression of a transcriptional regulator of adipogenesis. Of particular note is that, similar to paclitaxel, docetaxel and vincristine, which prevents microtubule assembly, facilitated transformation of Schwann cells into an immature state, characterized by increased expression of p75 and galectin-3. These results support the idea that taxanes (including paclitaxel)-mediated inhibition of cytoskeletal dynamics may induce dedifferentiation of Schwann cells, although further studies are needed to identify the underlying molecular mechanisms. The present findings allow us to consider that a unique phenomenon, such as paclitaxel-induced dedifferentiation of Schwann cells, is likely responsible for the disruption of cross talk between myelin-forming Schwann cells and axons, resulting in induction of CIPN after paclitaxel treatment.

In conclusion, the data presented herein suggest that cisplatin and oxaliplatin are directly cytotoxic to Schwann cells accompanied with mitochondrial dysfunction. By contrast, we show for the first time that paclitaxel causes dedifferentiation of Schwann cells. These phenomena may disrupt cross talk between myelin-forming mature Schwann cells and axons, thereby promoting peripheral neuropathy induced by the anti-cancer agents.

## Materials and Methods

### Drugs and chemicals

Cisplatin (LKT Laboratories, Inc., St Paul, MN, USA) and oxaliplatin (Wako Pure Chemical Industries, Osaka, Japan) were dissolved in phosphate-buffered saline (PBS, pH 7.4) at a concentration of 1 mM and distilled water at a concentration of 10 mM, respectively, prior to use. Paclitaxel (Sigma-Aldrich, St. Louis, MO, USA; 100 μM) was prepared in dimethyl sulfoxide (DMSO, Nacalai Tesque, Kyoto, Japan) and stored at −80 °C. Each drug solution was diluted in the appropriate culture medium to achieve the desired concentration before use.

### Animals

All experiments were performed according to the ethical guidelines of the Kyoto University Animal Research Committee. The protocol was approved by the Kyoto University Animal Research Committee (permission number: 2014–45, 2015–38, Med Kyo 15140 and Med Kyo 16067). All efforts were made to minimize the number of animals used and limit experimentation to only what was necessary. Pregnant Wistar/ST rats, male Wistar/ST rats (6–8 weeks old) and male C57BL/6 J mice (7–8 weeks old) were purchased from Japan SLC (Shizuoka, Japan). All animals were housed under a 12 h light-dark cycle at a constant ambient temperature (24 °C ± 1 °C) and humidity (55% ± 10%) and allowed access to food and water *ad libitum*. Schwann cells were prepared from the sciatic nerves of neonatal rats (1–4 days old).

### Culture of Schwann cells from the sciatic nerves of neonatal rats

Rat pups (post-natal day 1–4) were sacrificed by cervical dislocation, and the sciatic nerve was dissected quickly under sterile conditions. Following removal of the epineurium, the nerves were washed twice in ice-cold PBS and transferred to 24 well plates (Greiner Holding AG, Kremsmünster, Austria) containing 1 ml of culture medium (serum-free Advanced DMEM/F-12; Thermo Fisher Scientific, Waltham, MA, USA, supplemented with 0.292 mg/ml L-glutamine and 1% penicillin-streptomycin) containing 250 U/ml hyaluronidase type I-S (Sigma-Aldrich) and 160 U/ml collagenase type I (Sigma-Aldrich). After 2 h of incubation at 37 °C, tissue fragments were dissociated by trituration through 1 ml micropipette tips and the suspension was centrifuged at 300 × g for 5 min at room temperature. After re-suspension in maintenance medium (culture medium containing 2 μM forskolin (Sigma-Aldrich) dissolved in DMSO and supplemented with 20 ng/ml heregulin β-1 (Sigma-Aldrich) dissolved in 5% trehalose (Nacalai Tesque)), the cells were cultured on poly-L-lysine (PLL; Sigma-Aldrich)-coated 75 cm^2^ culture flasks. After 2 days of culture, Schwann cells were purified by magnetic activated cell separation with a monoclonal antibody against p75 neurotrophin receptor (Merck Millipore Co., Darmstadt, Germany), according to the manufacturer’s instructions (Miltenyi Biotec., Bergisch Gladbach, Germany). The cells were then cultured in PLL-coated 100 mm culture dishes containing Advanced DMEM/F-12 medium supplemented with 10% heat inactivated fetal bovine serum (FBS, Sigma-Aldrich), 1% penicillin-streptomycin, 0.292 mg/ml L-glutamine, 2 μM forskolin, and 10 ng/ml heregulin β-1. After 2 days, the cells were detached and incubated on ice with a mouse anti-CD90/Thy1 antibody (1:1000, Abcam, Cambridge, UK) diluted in serum-free culture medium containing 3% bovine serum albumin (BSA) to remove contaminating fibroblasts. After 2 h of culture, the suspension was centrifuged and the cells were incubated for an additional 1 h at 37 °C in rabbit complement-HLA-ABC (Thermo Fisher Scientific, Inc.) diluted 1:4 in serum-free culture medium containing 3% BSA. The suspension was centrifuged at 300 × g for 5 min, and the cells were washed twice with culture medium. The purified immature Schwann cells were then cultured under standard conditions (in maintenance medium) on tissue culture dishes or 10 mm coverslips (Matsunami Glass, Osaka, Japan) pre-coated with 0.1 mg/ml PLL. Cells were stained with DAPI, and the ratio of cells showing specific immunostaining for a Schwann cell phenotypic marker (S100β) or Thy-1 (contaminated cells) versus the total number of DAPI-positive cells was evaluated. The purity of the Schwann cells (based on S100β-immunolabeling) was initially 96%, but increased to 99.6% after purification.

### Schwann cell differentiation

At 4–7 days post-cell purification, the maintenance medium was removed and the cells were detached using 0.05% Trypsin-EDTA (Nacalai Tesque, Inc.). After detachment, the trypsin was deactivated by addition of medium supplemented with 10% FBS. To obtain differentiated Schwann cells, cells were cultured for 2 days in differentiation medium (serum-free Advanced DMEM/F-12 containing 1% penicillin-streptomycin, 0.292 mg/ml L-glutamine, 20 μM forskolin, and 20 ng/ml heregulin β-1) in 6 or 48 well plates or on coverslips pre-coated with 0.1 mg/ml PLL and 0.5 μg/ml laminin (Thermo Fisher Scientific, Inc.). Cells were exposed to anti-cancer agents 2 days after plating and culture in differentiation medium.

### Primary culture of DRG neurons from adult rats

After male rats (6–8 weeks old) were decapitated under deep anesthesia with 3% isoflurane (Nacalai Tesque), the skin was cut along the dorsal midline. The bone was then cut along the midline, and the spinal cord was removed with forceps by sliding them along the cord. The meninges were removed to visualize the DRG along the vertebral canal. The DRG were quickly removed one by one using forceps and transferred into ice-cold L-15 culture medium (Thermo Fisher Scientific). Next, dissected DRG from adult rats were transferred into a tube containing 1 ml of Hank’s balanced salt solution (137 mM NaCl, 5.4 mM KCl, 0.34 mM Na_2_HPO_4_, 0.44 mM KH_2_PO_4_, 5.6 mM D-glucose, and 2.4 mM 2-[4-(2-hydroxyethyl)-1-piperazinyl]ethanesulfonic acid, adjusted to pH 7.2 with NaOH) supplemented with 0.3% collagenase type II (Thermo Fisher Scientific, Inc.) and 0.4% dispase II (Thermo Fisher Scientific, Inc.) and incubated for 1 h at 37 °C with gentle shaking approximately every 15 min to ensure equal distribution of the DRG. After incubation, cells were mechanically dissociated using a pipette (triturating approximately 50 times). DRG neurons were purified by Percoll (Sigma-Aldrich) density gradient centrifugation. Percoll solutions (30% and 70%) were prepared in L-15 culture medium. A Percoll gradient was constructed by layering 3 ml of each Percoll solution into a centrifuge tube. Finally, 1 ml of DRG cell suspension was placed on top of the gradient and centrifuged at 1,800 × g for 15 min at room temperature. Intermediate fractions between each Percoll solution were collected. After washing with L-15 culture medium, cells were re-suspended in Neurobasal medium (Thermo Fisher Scientific) containing 2% B-27 supplement (Thermo Fisher Scientific), 2 mM L-glutamine, 1% penicillin-streptomycin, and 0.05 μg/ml nerve growth factor 2.5 S (Sigma-Aldrich). Purified DRG neuronal cells were cultured for 10 days on PLL/laminin-coated coverslips in Neurobasal medium (Fisherbrand Microscope Cover Glass, Thermo Fisher Scientific).

### Co-culture of Schwann cells and DRG neurons

Schwann cell/DRG neuron co-cultures were prepared as described previously^52^. Briefly, DRG were dissected from embryonic day 15 Wistar/ST rat pups and plated (2 × 10^5^ cells per well) in 24 well plates coated with PLL and laminin and containing Neurobasal medium supplemented with B27 and 100 ng/ml 2.5 S NGF. Non-neuronal cells were removed by treating the cultures with medium containing 5-fluorodeoxyuridine (FdUr) (Sigma-Aldrich). Ten days later, the cultures were switched to fresh medium without FdUr. Primary Schwann cells were obtained from the sciatic nerves of Wistar/ST rat pups (post-natal day 2). Contaminating fibroblasts were removed by treating the cells with 10 μM cytosine arabinoside for 48 h and by complement-mediated cytolysis using an anti-Thy1.1 antibody (Serotec) and rabbit complement (Cappel). Schwann cells were seeded onto PLL-coated plates and cultured in DMEM supplemented with 10% FBS, 2 μM forskolin, and 20 ng/ml rh-HRG-1 (Sigma). After the neural axons grew and expanded, Schwann cells (3 × 10^5^ cells per well) were then plated onto established neuronal cultures in DMEM supplemented with 10% FBS and 100 ng/ml 2.5 S NGF. Ten days later, the mixed culture of Schwann cells and neurons was treated with 50 μg/ml ascorbic acid to induce myelination.

### Viability of Schwann cells

Immature Schwann cells were seeded in 48 well plates. After reaching 70% confluence, cells were differentiated in differentiation medium. After 2 days of culture, cells were exposed to cisplatin (0.1–10 μM), oxaliplatin (0.1–10 μM), or paclitaxel (0.001–0.1 μM) for 1 or 2 days. The plated cells were then incubated at 37 °C for 45 min in 20 μl of MTT (Nacalai Tesque) solution (final concentration of 0.05 mg/ml). The medium was then removed, and the purple formazan crystals were dissolved in 150 μl of DMSO. An aliquot of 100 μl was extracted from each well and transferred to 96 well plates. The absorbance was then measured in a micro-plate reader (MAXline, Molecular Devices, Sunnyvale, CA, USA) at 560/630 nm. Cell viability was expressed as the absorbance value of drug-treated groups × 100/the absorbance value of the vehicle-treated group.

### Immunocytochemical analysis of cultured cells

Schwann cells, DRG neurons, and Schwann cell/DRG neuron co-cultures were cultured on PLL/laminin-coated coverslips. After differentiation or drug treatment, the culture medium was removed and the cells were washed with PBS and fixed with 4% paraformaldehyde in PBS for 20 min at room temperature. Cells were washed three times with PBS, blocked, and permeabilized for 30 min in 3% BSA in PBS containing 0.1% Tween 20. Consecutively, cells were incubated with mouse anti-CD90/Thy1 (1:1000, Abcam), rat anti-MBP (1:250, Merck Millipore), mouse anti-p75 (1:500, Merck Millipore), anti-GFAP (1:500, Merck Millipore), anti-MAP2 (1:500, Merck Millipore), or rabbit anti-S100 (1:1000, Agilent Technologies, Santa Clara, CA, USA) primary antibodies overnight at 4 °C. After washing three times with PBS, cells were incubated for 2 h at room temperature with appropriate secondary antibodies conjugated to Alexa Fluor™ 594 and/or Alexa Fluor™ 488 (1:200, Thermo Fisher Scientific). After washing, cultures were mounted using Vectashield containing DAPI (Vector Laboratories, Burlingame, CA, USA) and images were acquired under a laser scanning confocal microscope (Fluoview FV10i Confocal Microscope, Olympus Corporation, Tokyo, Japan). Next, 6–9 fields (2–3 fields × three independent samples) were selected randomly from each group and the immunoreactive signal intensity was measured using a computer-assisted system (Image J software, National Institute of Mental Health, Bethesda, MD, USA). The upper and lower threshold density ranges were adjusted to encompass and match the immunoreactivity to yield an image showing immunoreactive material as white pixels and non-immunoreactive material as black pixels. The density of the pixels within the threshold value representing immunoreactivity was then calculated. In each case, relative intensity (%) was expressed as the value for the drug-treated groups × 100/the value for the vehicle-treated group.

### Immunohistochemical analysis of mouse sciatic nerve sections

Male mice received an intraperitoneal (i.p.) injection of paclitaxel (4 mg/kg) or saline on Days 0, 3, 5, and 7 as described previously^53^. On the day after the last injection, mice were deeply anesthetized with pentobarbital (50 mg/kg, i.p.; Nacalai Tesque) and intra-cardially perfused with freshly prepared 4% paraformaldehyde in 0.1 M phosphate buffer (PB). Subsequently, bilateral sciatic nerves were quickly removed. Sciatic nerve sections were post-fixed in 4% paraformaldehyde for 4 h and permeated with 15% sucrose solution in 0.1 M PB for 24 h at 4 °C. The sciatic nerve sections were then frozen in an embedding compound (Sakura Fintek USA, Torrance, CA, USA) and stored at −80 °C until use. Frozen longitudinal segments of sciatic nerve were cut with a freezing cryostat (Leica CM 1850, Leica Microsystems, Wetzlar, Germany) (16 μm thick) and thaw-mounted on MAS-coated glass slides (Matsunami Glass Ind). The sections were then blocked in blocking buffer (PBS containing 0.1% Tween 20 and 5% normal goat serum, Vector Laboratories) for 1 h at room temperature and then incubated for 24 h at 4 °C with the following primary antibodies: mouse anti-p75 (1:500, Abcam) plus rabbit anti-MAP2 (1:250, Merck Millipore) or rat anti-GFAP (1:50, Merck Millipore), or rabbit anti-p75 (1:100, Abcam) plus mouse anti-galectin-3 (1:150, Abcam). The antibodies were rinsed away using PBS (three times) and incubated for 1.5 h at room temperature with appropriate secondary antibodies conjugated to Alexa Fluor™ 488 and/or 594 (1:200, Thermo Fisher Scientific). The slides were then cover-slipped with Vectashield. All images were acquired under a laser scanning confocal microscope (Fluoview FV10i Confocal Microscope, Olympus Corporation). Nine fields (three fields × three independent samples) were selected randomly per group, and the immunoreactive signal intensity was measured using a computer-assisted system (Image J). The images were analyzed, and immunoreactivity was calculated as described above.

### Western blotting

Schwann cells were homogenized in RIPA-lysing buffer (50 mM Tris-HCl buffer (pH 7.6), 150 mM NaCl, 1% Nonidet P-40, 0.5% sodium deoxycholate, protease inhibitor cocktail (Nacalai Tesque), and 0.1% sodium dodecyl sulfate (SDS)) using a sonicator (Q55 model, Qsonica, Newtown, CT, USA) and then centrifuged at 1000 × g at 4 °C for 10 min. The supernatants were collected, and protein concentrations were determined using a BCA Protein Assay Kit (Thermo Fisher Scientific). An aliquot of supernatant was diluted in NuPAGE^®^ LDS sample buffer (Thermo Fisher Scientific), and proteins were separated on a 3–12% SDS-polyacrylamide gradient gel. Proteins were transferred to a polyvinylidene difluoride (PVDF) membrane (Merck Millipore), which was then blocked for 1 h at room temperature in Blocking One solution (Nacalai Tesque) or blocking buffer comprising Tris-buffered saline (TBS) containing 5.0% Difco™ skim milk (Becton, Dickinson and Company, Franklin Lakes, NJ, USA) and 0.1% Tween 20. Next, the PVDF membrane was incubated with the following primary antibodies in 10% blocking one solution in TBS containing 0.1% Tween 20 (T-TBS) or in blocking buffer overnight at 4 °C: goat anti-actin (1:2500, Santa Cruz Biotechnology, Dallas, TX, USA), mouse anti-Krox20 (1:500, Abnova Corporation, Taipei, Taiwan), rabbit anti-Krox20 (1:200, Santa Cruz Biotechnology), mouse anti-MBP (1:1000, Abcam), goat anti-Oct6 (1:2000, Santa Cruz Biotechnology), or rabbit anti-Sox10 (1:1000, Abcam). The membrane was washed in T-TBS, followed by a 1 h incubation at room temperature with horseradish peroxidase (HRP)-conjugated anti-goat, anti-rabbit, anti-mouse, or anti-chicken IgG (Jackson ImmunoResearch, West Grove, PA, USA) antibodies diluted 1:2500 in T-TBS. The PVDF membrane was then washed in T-TBS. The antigen-antibody peroxidase complex was detected using enhanced chemiluminescence (Immobilon™, Merck Millipore) according to the manufacturer’s instructions, and images were generated using ImageQuant™ (LAS500, GE Healthcare Life-Sciences, Uppsala, Sweden). The intensity of each band was determined by Image J software and normalized against actin (loading control). Relative density (%) was expressed as the values for the drug-treated groups × 100/the value for the vehicle-treated group.

### Reverse transcription-polymerase chain reaction (RT-PCR) assay

Total RNA was extracted from Schwann cells using the SV Total RNA Isolation system (Promega, Madison, WI, USA), according to the manufacturer’s instructions. Purified total RNA was quantified in a spectrophotometer at 260 nm (NanoDrop 2000; Thermo Fisher Scientific). To prepare first strand cDNA, 0.2–1.0 μg of RNA was incubated in 40 μl of buffer containing a dNTP mixture, a RT random primer, and reverse transcriptase (High Capacity cDNA Reverse Transcription Kit, Thermo Fisher Scientific), according to the manufacturer’s instructions. Each target gene was amplified in a 50 μl PCR solution containing 2 mM MgCl_2_, 0.2 mM dNTP mix, and DNA polymerase (Blend Taq^®^, TOYOBO, Osaka, Japan) along with synthesized primers targeting MBP (sense: 5′-GAA GCC AGG ATT TGG CTA CG-3′, antisense: 5′-CAG AGC GGC TGT CTC TTC CT-3′; designed according to GenBank™ sequence accession number NM_001025291), Krox20 (sense: 5′-CTA CCC GGT GGA AGA CCT C-3′, antisense: 5′-AAT GTT GAT CAT GCC ATC TCC-3′; designed according to GenBank™ sequence accession number U78102.1), Sox10 (sense: 5′-TGC TAT CCA GGC TCA CTA CA-3′, antisense: 5′-CAG CTC AGT CAC ATC AAA GG-3′; designed according to GenBank™ sequence accession number NM_019193.2), p75 (sense: 5′-GCT GGG TTA CCA GCC TGA AC-3′, antisense: 5′-GCA GTG GAC TCG CTG CAT AG-3′; designed according to GenBank™ sequence accession number NM_012610), and glyceraldehyde 3-phosphate dehydrogenase (GAPDH, sense: 5′-GTT ACC AGG GCT GCC TTC TC-3′, antisense: 5′-TGA TGA CCA GCT TCC CAT TC-3′; designed according to GenBank™ sequence accession number NM_017008). Samples were heated to 94 °C for 2.5 min, 55 °C for 30 sec, and 72 °C for 1 min and cycled 30 times through 94 °C for 30 sec, 55 °C for 30 sec, and 72 °C for 1 min, with a final extension step at 72 °C for 3 min. The mixture was run on 1.5% agarose gels with the indicated markers (TOYOBO). The agarose gel was stained with ethidium bromide (Nacalai Tesque) and photographed under UV transillumination (PhotoDoc-It™ Imaging System, Analytik Jena AG, Jena, Germany).

### Quantitative analysis by real-time PCR

Extraction of total RNA from Schwann cells and preparation of cDNA were conducted as described above. cDNA was amplified in 20 μl of a PCR solution containing 10 μl of Power SYBR^®^ Green PCR Master Mix (Thermo Fisher Scientific) and primers targeting p75 (sense: 5′-GCT GGG TTA CCA GCC TGA AC-3′, antisense: 5′-GCA GTG GAC TCG CTG CAT AG-3′), galectin-3 (sense: 5′-GAC ATC GCC TTC CAC TTT AAC C-3′, antisense: 5′-GTC TTT CTT CCC TTC CCC AGT T-3′), and GAPDH (sense: 5′-GTT ACC AGG GCT GCC TTC TC-3′, antisense: 5′-TGA TGA CCA GCT TCC CAT TC-3′). PCR was performed using the StepOnePlus™ System (Thermo Fisher Scientific) under the following cycling conditions: 95 °C for 10 min and 60 °C for 1 min, followed by 40 cycles of 95 °C for 15 sec and 60 °C for 1 min. Fluorescence was detected after each extension step. GAPDH was used as a normalization control, and relative mRNA levels were calculated using a comparative C_t_ method and StepOnePlus™ software (Thermo Fisher Scientific).

### Mitochondrial transmembrane potential assay

The mitochondrial transmembrane potential (Δψ_m_) of Schwann cells was analyzed using the Δψ_m_-sensitive probe MitoTracker^®^ Red CM-H2XRos (Thermo Fisher Scientific) and fluorescence microscopy, according to the manufacturer’s instructions. Briefly, 48 h after treatment with anti-cancer agents, the culture medium was removed from cells and replaced with serum-free Advanced DMEM/F-12 containing 250 nM MitoTracker^®^ probe. The mixture was then incubated at 37 °C for 30 min. Cells were then washed three times with warm serum-free Advanced DMEM/F-12, fixed for 20 min with 4% paraformaldehyde, and viewed under a fluorescence microscope (Fluoview FV10i-W Confocal Microscope, Olympus). To count immunofluorescent-labeled cells, samples stained with MitoTracker^®^ Red CM-H2XRos were photographed using FV10i-SW at ×15 magnification. Twelve fields (four fields × three independent samples) were selected randomly per group, and immunofluorescent cells were counted. Finally, the total number of positive cells per 100 total cells was estimated.

### Statistical analysis

Data were analyzed using GraphPad Prism 5 (GraphPad Software, La Jolla, CA, USA) and expressed as the mean ± S.E.M. Differences between two groups were compared using Student’s t-test. Data from more than two groups were compared using one-way or two-way analyses of variance, followed by the Tukey’s multiple comparison test. In all cases, differences of *p* < 0.05 were considered statistically significant.

## Electronic supplementary material

Supplementary Information
