# Supplementary material for: Taxanes and platinum derivatives impair Schwann cells via distinct mechanisms
Source: Sci Rep. 2017 Jul 20;7:5947. doi: 10.1038/s41598-017-05784-1 (PMC5519765; doi:10.1038/s41598-017-05784-1)
Supplement: Supplementary file 1 — Supplementary Information [file 41598_2017_5784_MOESM1_ESM.pdf]

# Taxanes and platinum derivatives impair Schwann cells via distinct mechanisms

**Satoshi Imai[1,*,#], Madoka Koyanagi[1,*], Ziauddin Azimi[2,3,*], Yui Nakazato[1], Mayuna Matsumoto[1], Takashi Ogihara[1], Atsushi Yonezawa[1], Tomohiro Omura[1], Shunsaku Nakagawa[1], Shuji Wakatsuki[4], Toshiyuki Araki[4], Shuji Kaneko[2], Takayuki Nakagawa[1,#], Kazuo Matsubara[1]**

* Satoshi Imai, Madoka Koyanagi and Ziauddin Azimi contributed equally to this work.

[1]Department of Clinical Pharmacology and Therapeutics, Kyoto University Hospital, 54 Shogoin-Kawahara-cho, Sakyo-ku, Kyoto 606-8507, Japan.

[2]Department of Molecular Pharmacology, Graduate School of Pharmaceutical Sciences, Kyoto University, 46-29 Yoshida-Shimoadachi-cho, Sakyo-ku, Kyoto 606-8501, Japan.

[3]Department of Biochemistry, Faculty of Pharmacy, Kabul University, Jamal Mina, Aliabad, Kabul, Afghanistan.

[4]Department of Peripheral Nervous System Research, National Institute of Neuroscience, National Center of Neurology and Psyhicatry, 4-1-1 Ogawa-Higashi, Kodaira, Tokyo 187-8502, Japan.

**# Correspondence to:** Satoshi Imai, Ph.D. and Takayuki Nakagawa, Ph.D.

Department of Clinical Pharmacology and Therapeutics, Kyoto University Hospital, 54 Shogoin-Kawahara-cho, Sakyo-ku, Kyoto 606-8507, Japan.

Tel: +81-75-751-4560

E-mail: tknakaga@kuhp.kyoto-u.ac.jp

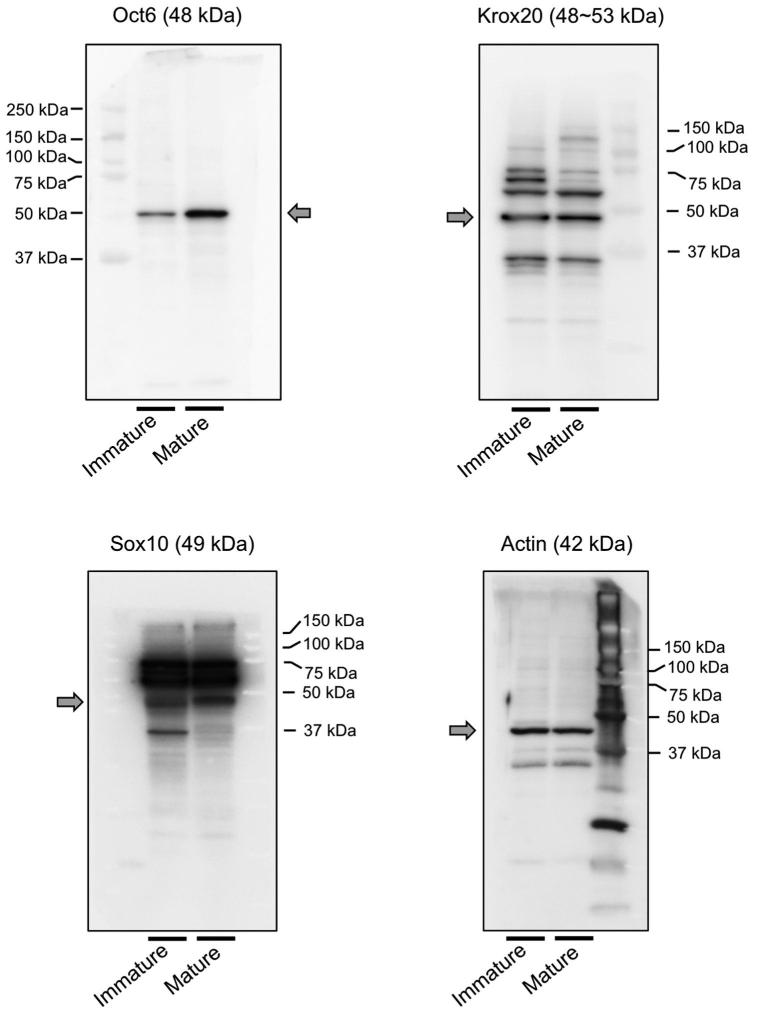


**Supplementary Fig. S1. Raw data of Western blot assay in Fig. 1A.** Western blot assay was performed as described in Materials and Methods. Briefly, Schwann cells were homogenized in RIPA-lysing buffer and then centrifuged at 1000 × g at 4°C for 10 min. The supernatants were collected, then an aliquot of proteins were separated on a 3–12% SDS-polyacrylamide gradient gel. Proteins were transferred to a polyvinylidene difluoride (PVDF) membrane (Merck Millipore), which was then blocked for 1 h at room temperature in Blocking solution. The PVDF membrane was incubated with the following primary antibodies: goat anti-actin (1:2500, Santa Cruz Biotechnology), mouse anti-Krox20 (1:500, Abnova Corporation), goat anti-Oct6 (1:2000, Santa Cruz Biotechnology), or rabbit anti-Sox10 (1:1000, Abcam). The membrane was washed in T-TBS, followed by a 1 h-incubation at room temperature with horseradish peroxidase (HRP)-conjugated anti-goat, anti-rabbit or anti-mouse IgG (Jackson ImmunoResearch) antibodies diluted 1:2500 in T-TBS. The antigen-antibody peroxidase complex was detected using enhanced chemiluminescence (Immobilon™, Merck Millipore). Grey arrows indicate a position of target proteins.

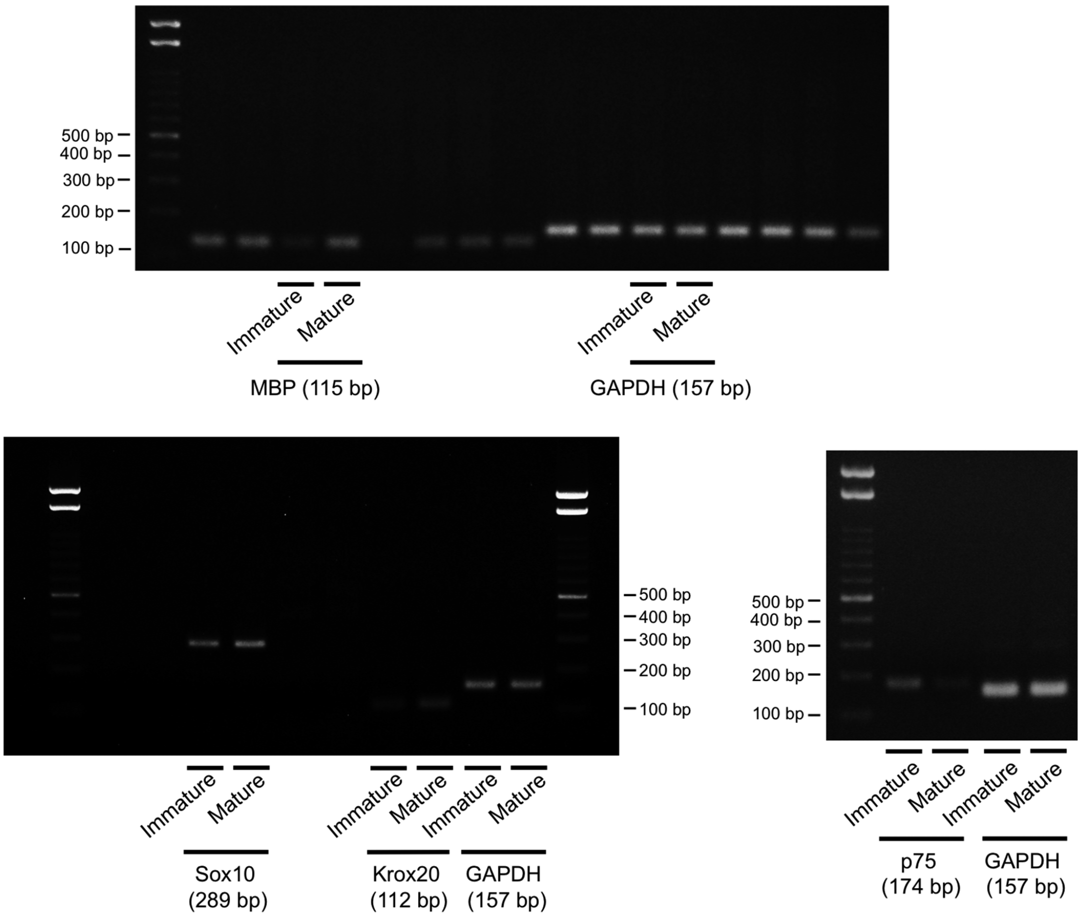


**Supplementary Fig. S2. Raw data of RT-PCR assay in Fig. 1C.** RT-PCR assay was performed as described in Materials and Methods. Briefly, total RNA was extracted from Schwann cells using the SV Total RNA Isolation system (Promega), according to the manufacturer's instructions. To prepare first strand cDNA, 0.2–1.0 µg of RNA was incubated in 40 µl of buffer containing a dNTP mixture, a RT random primer, and reverse transcriptase (High Capacity cDNA Reverse Transcription Kit, Thermo Fisher Scientific), according to the manufacturer's instructions. Each target gene was amplified in a 50 µl PCR solution containing 2 mM MgCl2, 0.2 mM dNTP mix, and DNA polymerase (Blend Taq®, TOYOBO) along with synthesized primers targeting MBP, Krox20, Sox10, p75, and glyceraldehyde 3-phosphate dehydrogenase (GAPDH). Samples were heated to 94°C for 2.5 min, 55°C for 30 sec, and 72°C for 1 min and cycled 30 times through 94°C for 30 sec, 55°C for 30 sec, and 72°C for 1 min, with a final extension step at 72°C for 3 min. The mixture was run on 1.5% agarose gels with the indicated markers (TOYOBO). The agarose gel was stained with ethidium bromide (Nacalai Tesque) and photographed under UV transillumination (PhotoDoc-It™ Imaging System, Analytik Jena AG).

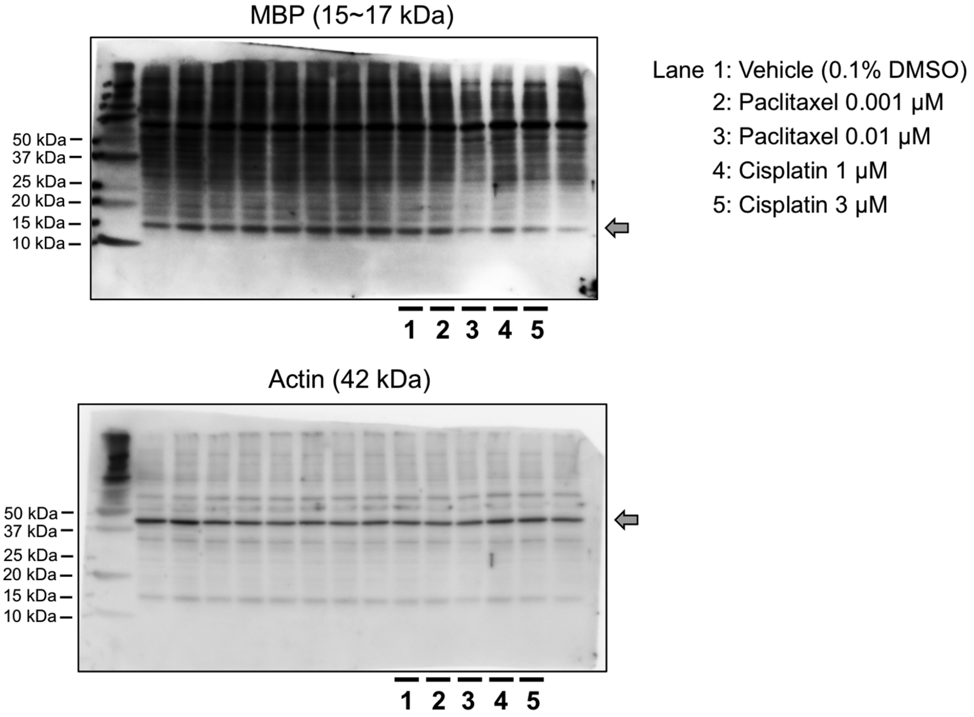


**Supplementary Fig. S3. Raw data of Western-blot assay in Fig. 3B.** Western-blot assay was performed as described in Materials and Methods. Briefly, Schwann cells were homogenized in RIPA-lysing buffer and then centrifuged at 1000 × g at 4°C for 10 min. The supernatants were collected, then an aliquot of proteins were separated on a 3–12% SDS-polyacrylamide gradient gel. Proteins were transferred to a polyvinylidene difluoride (PVDF) membrane (Merck Millipore), which was then blocked for 1 h at room temperature in Blocking solution. The PVDF membrane was incubated with the following primary antibodies: goat anti-actin (1:2500, Santa Cruz Biotechnology) or mouse anti-MBP (1:1000, Abcam). The membrane was washed in T-TBS, followed by a 1 h incubation at room temperature with horseradish peroxidase (HRP)-conjugated anti-goat or anti-mouse IgG (Jackson ImmunoResearch) antibodies diluted 1:2500 in T-TBS. The antigen-antibody peroxidase complex was detected using enhanced chemiluminescence (Immobilon™, Merck Millipore). Grey arrows indicate a position of target proteins. Lane 1: Vehicle (0.1% DMSO), Lane 2: Paclitaxel 0.001 μM, Lane 3: Paclitaxel 0.01 μM, Lane 4: Cisplatin 1 μM and Lane 5: Cisplatin 3 μM.

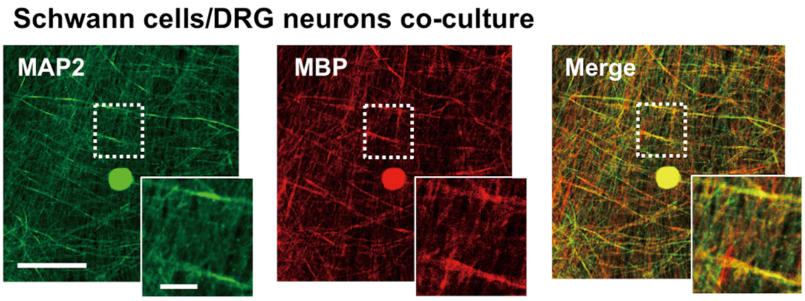


**Supplementary Fig. S4. co-cultures of Schwann cells and DRG neurons.** In vitro myelination was induced in a co-culture of DRG neurons and Schwann cells. Representative images of cultures immunostained for MAP2 (green) and MBP (red) are shown. Scale bar: 100 μm (25 μm in enlarged images).

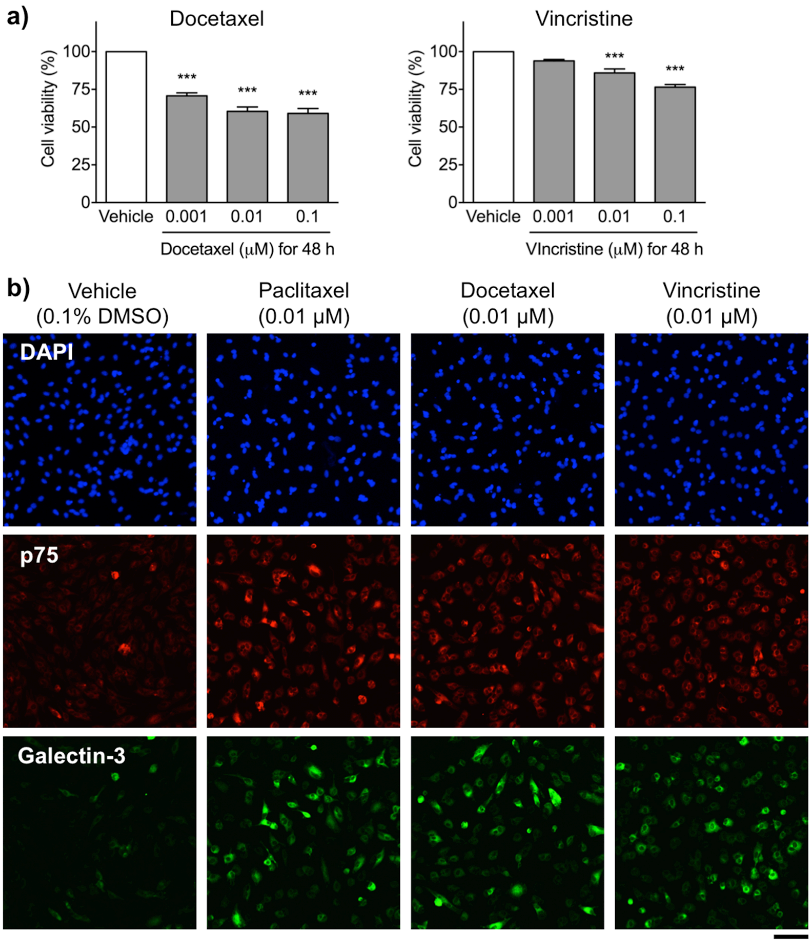


**Supplementary Fig. S5. Direct effects of docetaxel or vincristine on primary cultured Schwann cells obtained from the sciatic nerves of neonatal rat pups.** Vincristine sulfate (Wako Pure Chemical Industries) and docetaxel were dissolved in phosphate-buffered saline (PBS, pH 7.4) and dimethyl sulfoxide (DMSO), respectively, at the concentration of 1 mM. (**a**) Viability of Schwann cells after exposure to docetaxel or vincristine. At 2 days after culture in differentiation medium, Schwann cells were treated with vehicle (0.1% DMSO), docetaxel (0.001–0.1 μM) or vincristine (0.001–0.1 μM) for 48 h. Cell viability was measured in an MTT assay, and the results were expressed as a percentage relative to vehicle-treated cells. Each column represents the mean ± S.E.M. $n$ = 5. Statistical analysis was performed using one-way ANOVA, followed by the Tukey's multiple comparison test. ***$p$ < 0.001 *vs.* the vehicle-treated group. (**b**) Effect of paclitaxel, docetaxel or vincristine on expression of p75 and galectin-3 in primary cultured Schwann cells. Schwann cells were treated with vehicle (0.1% DMSO), paclitaxel (0.01 μM), docetaxel (0.01 μM) or vincristine (0.01 μM) for 48 h. Immunofluorescent staining for DAPI (blue), p75 (red) and GFAP (green). Scale bars: 50 μm.

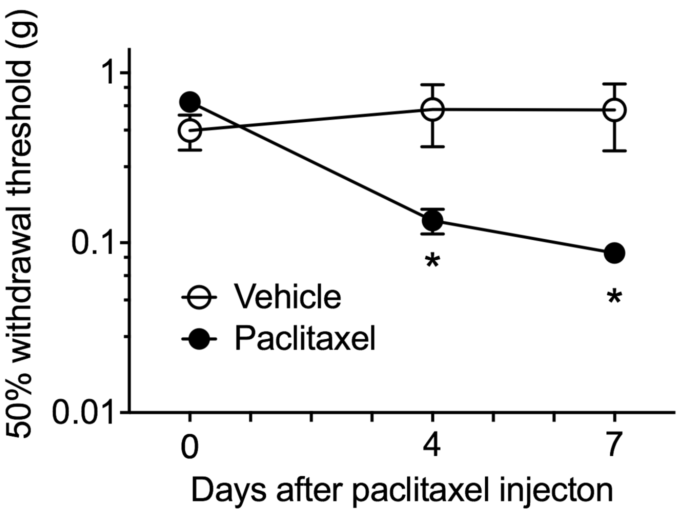


**Supplementary Fig. S6. Repeated injection of paclitaxel induced mechanical allodynia in mice.** Male C57BL/6J mice (7–8 weeks old) were purchased from Japan SLC. Mice were injected with paclitaxel (4 mg/kg; i.p.) once daily on day 0, 3, 5, and 7. Mechanical sensitivity was assessed by the up-down method using calibrated von Frey filaments. Mice were acclimatized on a metal mesh floor in small cylinders for 2 h. The mechanical sensitivity was evaluated using a set of seven calibrated von Frey filaments (0.008, 0.02, 0.04, 0.07, 0.16, 0.4, and 1.0 g; Stoelting) that were applied to the plantar surface of the hind paw until the filament bent slightly for a few seconds. The 0.16 g filament was always the first stimulus. When there was a positive response, such as flicking or lifting, the next lower filament was applied, and when there was no response, the next higher filament was used. The 50% paw withdrawal threshold value was calculated from five consecutive responses (Dixon, Annu Rev Pharmacol Toxicol 20, 441-462, 1980; Chaplan et al. J Neurosci Methods 53, 55-63, 1994). Mechanical sensitivity was assessed before and 4 and 7 days after the first injection. Each point represents the mean ± S.E.M. $n = 4–8$. Statistical analysis was performed using the Statistical analysis was performed using repeated measured two-way ANOVA, followed by the Bonferroni *post hoc* test. $*p < 0.05$ vs. the vehicle-treated group.
